# Unraveling the mystery of Tacaribe virus

**DOI:** 10.1128/msphere.00605-24

**Published:** 2024-09-18

**Authors:** Tony Schountz

**Affiliations:** 1 Department of Microbiology, Immunology and Pathology, Center for Vector-borne Infectious Diseases, College of Veterinary Medicine and Biomedical Sciences, Colorado State University, Fort Collins, Colorado, USA; University of Michigan, Ann Arbor, Michigan, USA

**Keywords:** *Artibeus*, Tacaribe virus, TCRV, bats, reservoir

## Abstract

Tacaribe virus (TCRV) was first isolated in the mid-1950s from several *Artibeus* species bats in and around Port of Spain, Trinidad and Tobago. Since that time, debate has persisted whether artibeus bats serve as reservoir hosts of the virus or whether infection of the bats was an incidental spillover event from another, unidentified reservoir host. Complicating the issue is that the only TCRV isolate routinely used, TRVL-11573, had been passaged in suckling mice and likely accumulated mutations that altered its biology. Recent fieldwork has now identified two distinct genomes of TCRV in apparently healthy artibeus bats sampled in Brazil and the Dominican Republic (C. Fischer, M. H. A. Cassiano, W. R. Thomas, L. M. Dávalos, et al., mSphere e00520-24, 2024, https://doi.org/10.1128/msphere.00520-24). Together, these works suggest that artibeus bats are natural reservoirs of TCRV and that the virus has a wide geographic distribution in the Americas.

## COMMENTARY

In March 1956, Wilbur Downs found his cat clutching a great fruit-eating bat (*Artibeus lituratus*) in the yard of his home in Port of Spain, Trinidad and Tobago (E. Tikasingh, 2011, personal communication). At the time, Downs was the director of the Trinidad Regional Virus Laboratory (TRVL), which was supported by the Rockefeller Foundation to identify infectious agents of tropical diseases. Downs suspected the bat had rabies, but histology failed to identify Negri bodies in the brain, thereby excluding rabies virus infection (1). He intracranially inoculated 12 suckling mice with brain and salivary gland homogenates from this bat and by several days later 10 of the mice had died. The virus was named Tacaribe virus (TCRV; *Mammarenavirus tacaribeense*) after a regional tribe, and the brain isolate was given the designation TRVL-11573. The virus, which was later determined to be an arenavirus, was subsequently isolated from 10 other artibeus bats of two species during the next 2 years, including what likely was misidentified flat-faced fruit-eating bats (*Artibeus planirostris*) (2), and one mosquito pool. Unfortunately, none of the other isolates remain; all were lost over the intervening decades. TRVL-11573 was intracranially passaged 22 times in suckling mice (1), which may have led to neurotropism. Sequencing of subsequent passaged virus identified several errors and mutations in TRVL-11573’s genome (3). More recent efforts to isolate TCRV from bats in Trinidad failed; however, high rates of seroprevalence to TCRV antigen in Trinidadian bats suggest that TCRV or similar arenaviruses still circulate in bats of Trinidad (4). No natural infections of humans have been reported; however, at least one laboratory infection with TRVL-11573 has occurred that caused febrile illness and seroconversion (G. Gard, 2009, personal communication), suggesting TCRV may have zoonotic potential.

No other TCRV isolates were made until the early 2010s, when the virus was isolated from a pool of lone star ticks (*Amblyomma americanum*) collected in central Florida (5). This was highly unexpected because arenaviruses are not associated with ticks and artibeus bats are not found in central Florida. The vertebrate source of this isolate is still unknown, but because mammarenaviruses with known hosts are hosted by rodents, it may have originated from a rodent.

Subsequent testing of more than 1,000 bats sampled in Brazil not only identified TCRV infection of four artibeus bats but also a novel, closely related arenavirus in samples collected from 12 Seba’s short-tailed fruit bats (*Carollia perspicillata*) named Tietȇ virus (6), suggesting New World fruit bats may host other TCRV serogroup arenaviruses. This work showed that arenavirus infection of the bats was likely systemic, with multiple organs of some bats containing viral RNA. In total, less than 2% of the sampled bats were infected, perhaps due to viral clearance.

Recently in *mSphere*, Fischer et al. (7) identified a complete but divergent TCRV genome from archived Jamaican fruit bat (*Artibeus jamaicensis*) transcriptome data collected in the Dominican Republic in 2014. Fortuitously, necropsy samples from this bat had been preserved, and the authors subsequently identified TCRV sequences in multiple tissues. These exhibited 83%–86% nucleotide and 92%–94% amino acid identity to TRVL-11573 and TCRV detected in flat-faced fruit-eating bats sampled in Brazil.

The accumulated body of evidence suggests that multiple species of artibeus bats serve as reservoir hosts of TCRV and that the virus has a wide geographic distribution in the tropical Americas. But many questions remain. Downs intramuscularly inoculated several wild-caught artibeus bats of unknown infection history with TCRV but none of them became obviously ill (1). Serum from one of the bats protected mice from infection; however, complement fixation testing of the serum did not exhibit reactivity, perhaps reflecting a lack of guinea pig complement binding to artibeus bat IgG. It is not clear whether Downs used TRVL-11573 or one of the other isolates for the challenge study, nor is it clear what virus passage level was used. In contrast, later studies using Jamaican fruit bats from a closed, captive breeding colony showed that high-dose intranasal and subcutaneous inoculation with TRVL-11573 caused fatal disease with conspicuous neurological manifestations, despite evidence of both antiviral and adaptive immune responses (8, 9). TRVL-11573 may have adapted to cause neuropathology due to multiple intracranial passages in neonatal mice. Similar to what Downs reported, most of the surviving bats in this study did not seroconvert, but the three that did, did so between 21 and 45 days post-infection and with very low neutralizing titers. Because the serostatus of the bats used in the Downs study was unknown, it is possible that the bat that had antibodies may have been infected prior to capture and that experimental challenge induced an anamnestic antibody response.

Experimental infection of Jamaican fruit bats with wild-type TCRV may be more informative. Although one other isolate has been made, it is from a tick pool (5) and has a higher sequence identity to TRVL-11573 than to the Brazilian or Dominican Republic TCRV genomes (3). At least one infectious clone has been made from TRVL-11573’s genome (10), but a low passage isolate from an artibeus bat or infectious clone from the genome described in Fischer et al. (7) is likely to be more relevant for a repeat study in Jamaican fruit bats.

The isolation of TCRV from mosquitos in Trinidad in the 1950s and ticks in Florida in 2012 suggests that the virus may use arthropods as vectors, resulting in viremia in vertebrate hosts. The difficulty with the Florida isolate is that it is unlikely to be from an artibeus bat because these bats are found only as far north as the Florida Keys. It may be that rodents or insectivorous bats may also harbor TCRV in central Florida; however, there are no reports of sampling these species to determine whether TCRV is circulating in vertebrates.

TCRV’s natural hosts are likely artibeus bats, with at least three species found to harbor divergent viruses. TCRV is phylogenetically related to the South American hemorrhagic fever arenaviruses (11); thus, it is important to reevaluate TCRV’s potential to infect humans using the viruses that were detected in the Dominican Republic and Brazil. Artibeus bats are frequently peridomestic, thus raising the apparent risk of spillover to humans.
